# One hundred years of climate change in Mexico

**DOI:** 10.1371/journal.pone.0209808

**Published:** 2020-07-16

**Authors:** Angela P. Cuervo-Robayo, Carolina Ureta, Miguel A. Gómez-Albores, Anny K. Meneses-Mosquera, Oswaldo Téllez-Valdés, Enrique Martínez-Meyer

**Affiliations:** 1 Departamento de Zoología, Instituto de Biología, Universidad Nacional Autónoma de México, Ciudad de México, México; 2 Comisión Nacional para el Conocimiento y Uso de la Biodiversidad (Conabio), Ciudad de México, México; 3 Cátedras-Departamento de Ciencias Atmosféricas, Centro de Ciencias de la Atmósfera, Universidad Nacional Autónoma de México, Ciudad de México, México; 4 Instituto Interamericano de Tecnología y Ciencias del Agua, Universidad Autónoma del Estado de México, Toluca, Estado de México, México; 5 Facultad de Estudios Superiores Iztacala, Unidad de Biotecnología y Prototipos, Laboratorio de Recursos Naturales, Universidad Nacional Autónoma de México, Tlalnepantla, México; University of Oregon, UNITED STATES

## Abstract

Spatial assessments of historical climate change provide information that can be used by scientists to analyze climate variation over time and evaluate, for example, its effects on biodiversity, in order to focus their research and conservation efforts. Despite the fact that there are global climatic databases available at high spatial resolution, they represent a short temporal window that impedes evaluating historical changes of climate and their impacts on biodiversity. To fill this gap, we developed climate gridded surfaces for Mexico for three periods that cover most of the 20^th^ and early 21^st^ centuries: *t*_*1*_-1940 (1910–1949), *t*_*2*_-1970 (1950–1979) and *t*_*3*_-2000 (1980–2009), and used these interpolated surfaces to describe how climate has changed over time, both countrywide and in its 19 biogeographic provinces. Results from our characterization of climate change indicate that the mean annual temperature has increased by nearly 0.2°C on average across the whole country from *t*_*2*_-1970 to *t*_*3*_-2000. However, changes have not been spatially uniform: Nearctic provinces in the north have suffered higher temperature increases than southern tropical regions. Central and southern provinces cooled at the beginning of the 20^th^ century but warmed consistently since the 1970s. Precipitation increased between *t*_*1*_-1940 and *t*_*2*_-1970 across the country, more notably in the northern provinces, and it decreased between *t*_*2*_*-*1970 and *t*_*3*_*-*2000 in most of the country. Results on the historical climate conditions in Mexico may be useful for climate change analyses for both environmental and social sciences. Nonetheless, our climatology was based on information from climate stations for which 9.4–36.2% presented inhomogeneities over time probably owing to non-climatic factors, and climate station density changed over time. Therefore, the estimated changes observed in our analysis need to be interpreted cautiously.

## Introduction

Climate change has been recognized as one of the major drivers of socio-environmental disruption in recent years [1,2] due to its strong effect on demographic, geographic and ecosystem processes [1,3–6]. Climate change acts in synergy with other environmental disruptive factors, such as habitat loss, pollution, overexploitation, and invasive species [7,8]. Currently, climate change studies in biodiversity have strongly focused on future projections with less attention to current and recent historical climate change impacts that could provide valuable information to guide conservation efforts.

The climate has been changing globally and non-uniformly since the mid-20^th^ century. Patterns of climate change are dynamic and highly heterogeneous across the planet: global mean surface temperature has increased around 0.85°C in the last 130 years, and since the 1970s, precipitation has generally increased in latitudes beyond 30° and decreased in the tropics [9,10,11].

One of the main inputs for regional or global climate change assessments are interpolated climate surfaces at a high spatial resolution of both present-day/historical climate and downscaled surfaces of future climate projections [12]. High-resolution gridded climate surfaces (referred to as “climate surfaces” from here on) for global land areas have been useful for assessing how climate change affects biodiversity [13]. Although global databases, such as *WorldClim* and *Climond*, are freely available [12,14], these databases present some shortcomings that impede evaluating the historical climate change and their impacts on biodiversity; for example, they span only a single period of time in the late 20^th^ century. Furthermore, the quality and reliability of global databases are compromised in many regions because the data from weather stations used for producing global interpolations generally come from open, public databases, whereas in some places–such as Mexico–there is more information available, but needs to be requested. Also, there is more time and effort devoted to detecting and correcting errors in databases if people are focused on a particular region than worldwide; for example, reconciling location (coordinates) with elevation of stations has been more thoroughly done at regional levels [14,15]. Until now, gridded climate surfaces for Mexico are only readily available for single time slices [15–17], so they are not suitable for evaluating temporal climatic changes. Regardless, time series information at the level of climate stations is available for climate change analysis [18,19], although some regions across the country lack adequate spatial coverage.

One option to overcome this limitation is to analyze data from climatic surfaces at different periods from the recent past and the present in an attempt to understand where, when and to what degree the climate has been changing, in an effort to evaluate its environmental and social implications [2,20–28]. For instance, combining data of climate surfaces with species inventories can be useful for detecting drivers of change in biological communities [28,29] and responses of biodiversity to climate change [30]. Historic climate surfaces can be used to identify areas that have been under the effect of climate change for some time to analyze the response and resilience of natural and social subsystems [31]. Furthermore, coupling this information with future projections can help to inform decision-makers [32–34]. This approach has been successful in Australia, where historical climate data facilitated conservationists to detect recent range shifts in bird communities that may be informative for future biotic responses [35].

To date, most studies evaluating the impacts of climate change on biodiversity have focused, first, on future projections rather than under a retrospective approach, and second, on individual species and ecosystems [1], and not at other organizational levels [36]. A more inclusive approach would be to assess climate change impacts in biogeographic units, which represent natural spatial areas that integrate physiographic, evolutionary and ecological features of biodiversity [37]. However, the impacts of climate change at this level have yet to be performed [32].

To fill this gap, we developed average historical climate surfaces for Mexico, for three periods covering most of the 20^th^ and early 21^th^ centuries: *t*_*1*_-1940 (1910–1949), *t*_*2*_-1970 (1950–1979) and *t*_*3*_-2000 (1980–2009). We used these surfaces to describe historical climate change countrywide and in the 19 biogeographic provinces of Mexico.

## Materials and methods

### Climate data

**Fig 1:**
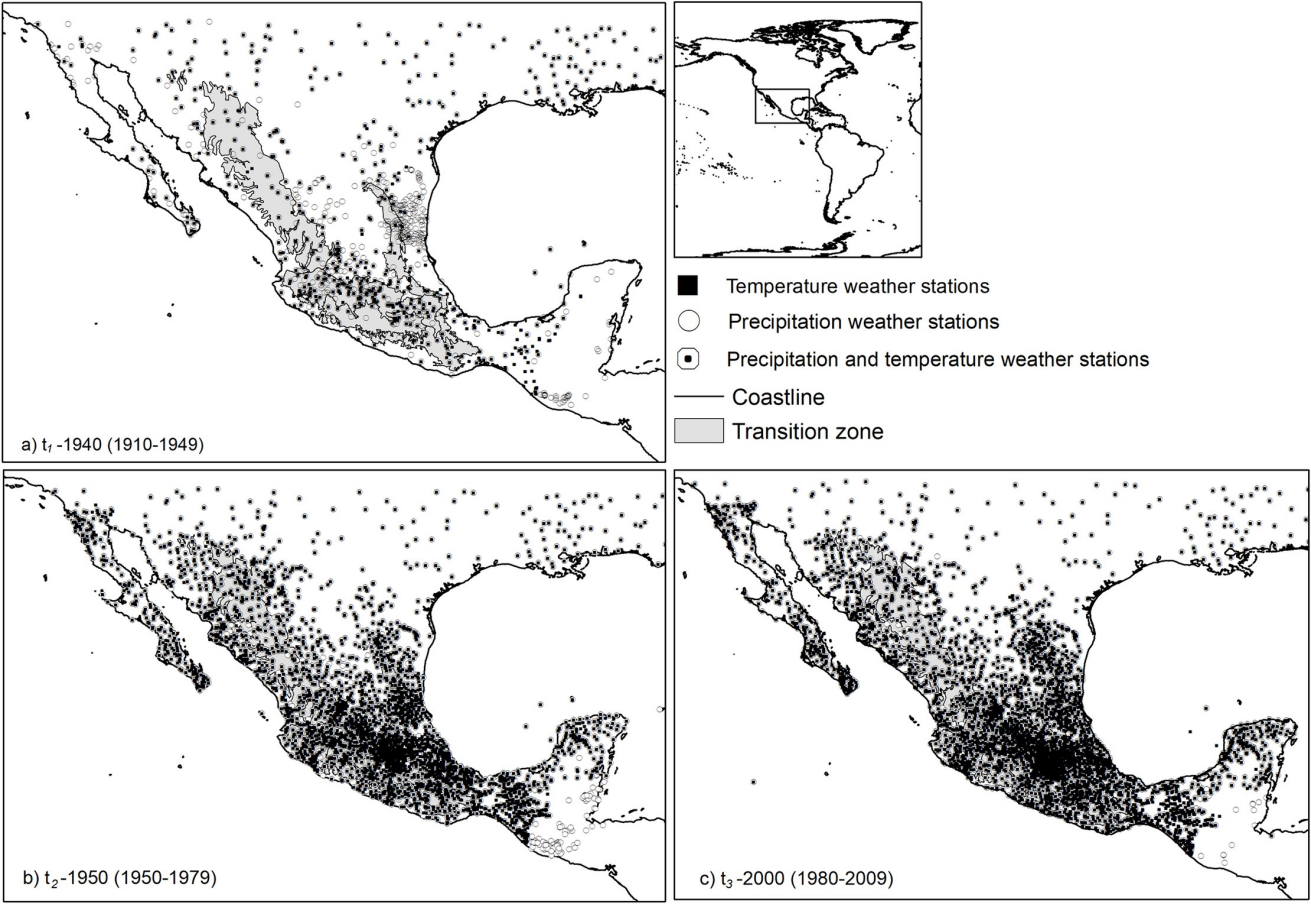
Location of weather stations used to generate climate surfaces of precipitation, maximum and minimum temperature for three periods: a. *t*_*1*_-1940 (1910–1949), b. *t*_*2*_-1970 (1950–1979) and c. *t*_*3*_*-*2000 (1980–2009). Shaded areas represent mountainous regions above 1500 masl. Stations used were not corrected for inhomogeneities and densities vary between periods and climate variables. For *t*_*1*_-1940 we used 803 stations for precipitation and 500 for temperature, for *t*_*2*_-1970, we used 3411 and 3670 and in *t*_*3*_*-*2000, 3870 and 4200 for precipitation and temperature, respectively.

We analyzed monthly minimum and maximum temperature and accumulated rainfall data gathered from weather stations from the National Meteorological Office that were previously organized by Cuervo-Robayo [17], to derive monthly mean climate surfaces for three periods: *t*_*1*_-1940 (1910–1949), *t*_*2*_-1970 (1950–1979) and *t*_*3*_*-*2000 (1980–2009). We selected these periods based on previous global [38,39] and regional [19] climate change analysis and we also considered the number of stations available for each period [40]. For *t*_*1*_*-*1940, we used a 40-year period, instead of 30 years as in the other time slices, due to the limited number of stations available. Because interpolation estimations are normally poor toward the edges of a region, the analysis should expand beyond the limits to the target region. Consequently, we included weather stations from southern portions of the United States, northern Belize and Guatemala. Data from the U.S. were gathered from the United States Historical Climatology Network (USHC: http://cdiac.ornl.gov/epubs/ndp/ushcn/access.html), and data from Belize and Guatemala were gathered through the FAOCLIM 2.0 software (http://www.fao.org/nr/climpag/pub/en1102_en.asp) and the National Climatic Data Center (http://www.ncdc.noaa.gov data were organized and averaged into their corresponding periods using the “Structuration” extension from the Integrated Water Resources Management tool implemented in Idrisi Selva software, which is freely available at http://idrisi.uaemex.mx [41]. For the period *t*_*1*_-1940, weather stations were located mainly in central Mexico, in the states of Guanajuato, Jalisco, Michoacán, and México. For the other two time periods, weather stations distributed throughout the country, mainly at or near densely populated areas (Fig 1).

We used ANUSPLIN software version 4.37 [42] to generate countrywide continuous climate surfaces for the continental area (i.e., excluding oceans). This program uses the thin-plate smoothing splines interpolation technique, which integrates climatic and topographic data for making spatial climatic estimations, thus performing better than other interpolation methods [43,44]. Nonetheless, methods that incorporate expert knowledge, e.g. PRISM [45], sometimes perform better to capture features, such as rain shadow, which can be advantageous in complex geographical regions. Finally, we only used climate data from stations that operated for more than 10 years (S1) in at least one variable (temperature or precipitation). We used a second-order spline with three independent variables (latitude, longitude and elevation) [17]. The value of the smoothing parameter was determined by minimizing a measure of the predictive error of the fitted surface given by the generalized cross-validation (GCV). For precipitation, we used a square-root transformation that reduces positively skewed values and ignores all negative values in precipitation data [42,46]. It also applies more smoothing to large precipitation values and less smoothing to small precipitation data values [47]. Given that we had more than 2000 stations for *t*_*2*_-1970 and *t*_*3*_-2000, we used SPLINB, as recommended by Hutchinson [46], and used SELNOT to select a set of knots to reduce the complexity of the fitted spline. For *t*_*1*_-1940 we used SPLINA, which uses all available stations [46,47].

ANUSPLIN produces a list of the stations with the largest data residuals that denote fitting errors. With this list, Cuervo-Robayo et al. [17] corrected the geographic position for 100 erroneous stations by using online gazetteers and Google Earth; however, for the two later periods, we eliminated about 20 stations that maintained high residual values (< 1) during the fitting process, without increasing error in the diagnostic statistics. This residual error may have resulted from the lack of a homogenization process in the stations’ data. We assessed the accuracy of the fitted surfaces by examining ANUSPLIN diagnostic measures [42]. The signal indicates the degrees of freedom associated with the surfaces, which reflects the complexity of the surface and varies between a small positive integer and the number of stations used to generate the surface [46,48]. Hutchinson & Gessler [46] suggested that the signal should not be greater than about half the number of data points. Models with a signal below this threshold tend to be more robust and reliable in regions where data are scarce [49]. We also examined the root mean square error (RTMSE) and the square root of the GCV (RTGCV). RTMSE is an optimistic assessment of predictive error because some interpolation error is removed, therefore the true error is somewhere between the RTMSE and the RTGCV error [42]. Gridded climate surface (i.e., gridpoints) of land areas were generated with the function LAPGRD, using a 30-arc second of spatial resolution digital elevation model (https://lta.cr.usgs.gov/GTOPO30). As an additional output, we derived 19 bioclimatic variables for each period with the *dismo* library [50] of R software [51]. These variables included annual, quarterly and monthly summaries of temperature and precipitation that represent more biologically meaningful variables than the original climate surfaces and have been widely used in several studies of climate change impacts on species and ecosystems [12]. For *t*_*1*_-1940, we used 803 stations for precipitation and 500 for minimum and maximum temperatures. For *t*_*2*_-1970, the number of stations was 3411 and 3670 for precipitation and temperature, respectively. For *t*_*3*_-2000, the number of stations was 3870 and 4200 for precipitation and temperature, respectively (Fig 1). Across the country, 47% of the weather stations are located in mountainous regions above 1500 masl; the remaining 63% are located in the lowlands. Data used for the analysis are available upon request, and the original dataset is available at the National Meteorological Office (http://smn.cna.gob.mx/es/, S1 File). Monthly climate surfaces and bioclimatic variables for each period are freely available (see URLs in Table 1).

**Table 1:** URLs for downloading monthly gridded surfaces of maximum temperature, minimum temperature and precipitation, and 19 bioclimatic parameters. Metadata can be accessed through the metadata tab.

Variable	Period	url
Monthly maximum temperature (*MaxT*)	*t*_*1*_-1940 (1910–1949)	http://geoportal.conabio.gob.mx/metadatos/doc/html/tm191049gw.html
	*t*_*2*_-1970 (1950–1979)	http://geoportal.conabio.gob.mx/metadatos/doc/html/tm195079gw.html
	*t*_*3*_*-*2000 (1980–2009)	http://geoportal.conabio.gob.mx/metadatos/doc/html/tm198009gw.html
Monthly minimum temperature (*MinT*)	*t*_*1*_-1940 (1910–1949)	http://geoportal.conabio.gob.mx/metadatos/doc/html/tmi191049gw.html
	*t*_*2*_-1970 (1950–1979)	http://geoportal.conabio.gob.mx/metadatos/doc/html/tmi195079gw.html
	*t*_*3*_*-*2000 (1980–2009)	http://geoportal.conabio.gob.mx/metadatos/doc/html/tmi198009gw.html
Monthly precipitation (*Ppt*)	*t*_*1*_-1940 (1910–1949)	http://geoportal.conabio.gob.mx/metadatos/doc/html/p19101949gw.html
	*t*_*2*_-1970 (1950–1979)	http://geoportal.conabio.gob.mx/metadatos/doc/html/p19501979gw.html
	*t*_*3*_*-*2000 (1980–2009)	http://geoportal.conabio.gob.mx/metadatos/doc/html/p19802009gw.html
19 Bioclimatic variables (*Bio*)	*t*_*1*_-1940 (1910–1949)	http://geoportal.conabio.gob.mx/metadatos/doc/html/b19101949gw.html
	*t*_*2*_-1970 (1950–1979)	http://geoportal.conabio.gob.mx/metadatos/doc/html/b19501979gw.html
	*t*_*3*_*-*2000 (1980–2009)	http://geoportal.conabio.gob.mx/metadatos/doc/html/b19802009gw.html

In order to evaluate differences between periods, we performed a discriminant analysis on deseasoned climatic surfaces using the Statistica software version 10 [52]. Deseasoning is recommended to eliminate seasonality from the analysis that may obscure temporal changes. To do so, we calculated standardized anomalies (*z*-scores) for each month of the three periods using the Deseason Panel of the Earth Trends Modeler (ETM) of Idrisi Selva [53]. We also evaluated if the difference in the number of weather stations between *t*_*2*_-1970 and *t*_*3*_-2000 had an effect on the climate surfaces; we did not compare *t*_*1*_-1940 against the other two periods because station instrumentation and especially density was quite different for the first period from that of the last two periods [40]. In order to compare *t*_*2*_-1970 and *t*_*3*_-2000, we created a set of climate surfaces in which we used the same number of weather stations for both periods. We found that differences between *t*_*2*_-1970 and *t*_*3*_-2000 were not due to the number of stations, thus we included all available stations for the second and third periods, mainly because a larger number of stations improve model signal [17,54].

### Biogeographic provinces of Mexico

**Fig 2:**
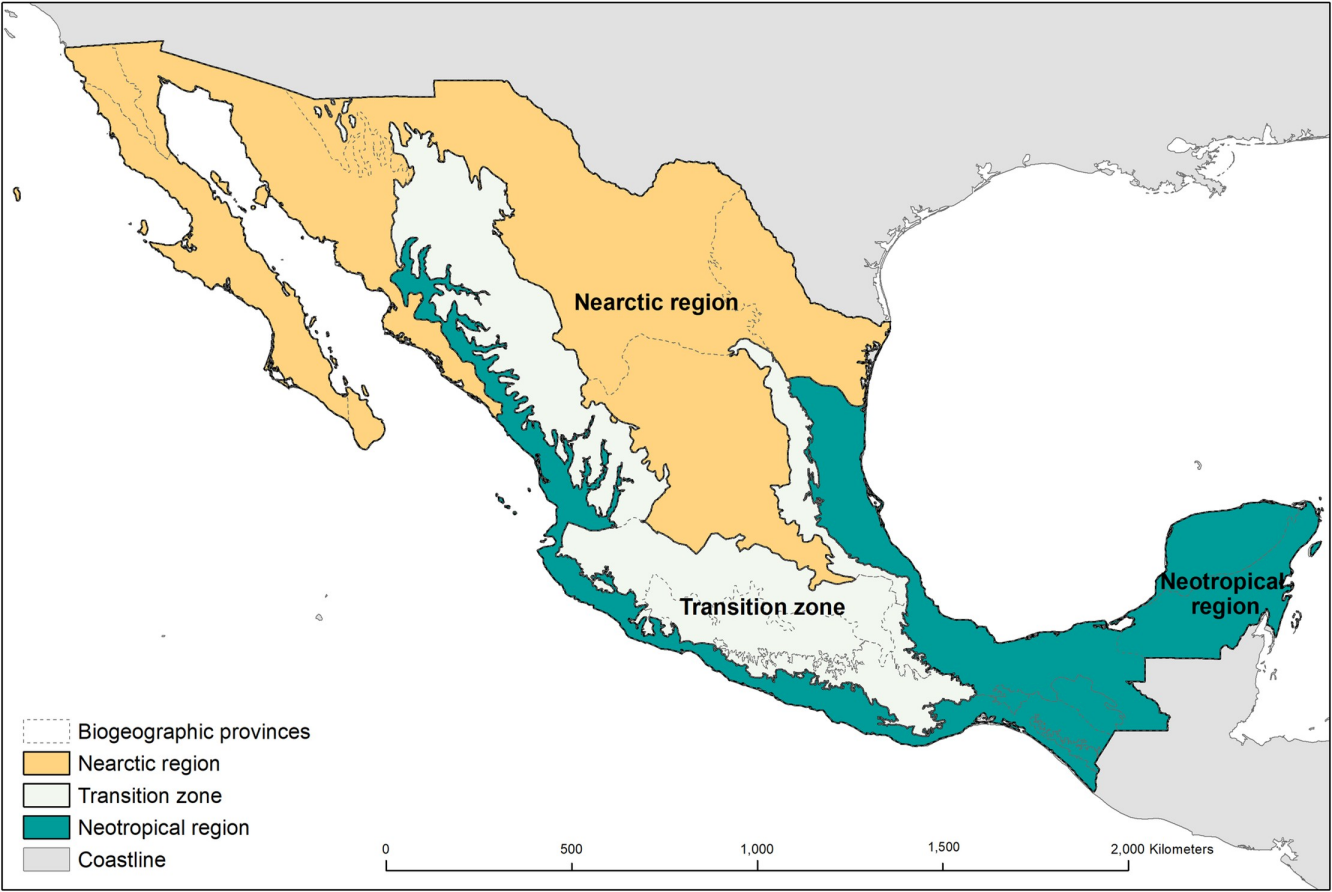
Biogeographic regions and provinces of Mexico: Nearctic and Neotropical regions, and the transition zone. Reprinted from [57] under a CC BY license, with permission from [CONABIO], original copyright [1997].

Mexico is recognized as a megadiverse country [55]. Its extraordinary biodiversity is due to its high environmental heterogeneity and the confluence of the Nearctic and Neotropical biogeographic regions connected by a transition zone (Fig 2) [56]. The Nearctic region encompasses the arid subtropical areas of the north and includes the provinces of: California, Baja California, Del Cabo, Sonorense, Altiplano Norte, Altiplano Sur, and Tamaulipeca. The Neotropical region covers the humid and sub-humid tropical areas of the south, including the provinces: Costa del Pacífico, Golfo de México, Depresión del Balsas, Oaxaca, Altos de Chiapas, Soconusco, Yucatán, and Petén. The Transition Zone includes the mountainous provinces in the central portion of the country: Sierra Madre Oriental, Sierra Madre Occidental, Eje Volcánico, and Sierra Madre del Sur [56,57].

A map of the biogeographic provinces of Mexico (Fig 2) was obtained from the *Comisión Nacional para el Conocimiento y Uso de la Biodiversidad* [57]. This regionalization was based on the distributional pattern of four taxonomic groups (vascular plants, amphibians, reptiles, and mammals) and the main morpho-tectonic features of Mexico [56]. Each unit represents a relatively homogeneous area with high levels of endemic species sharing similar historical, physiographic, climatic, edaphic, and vegetation features [57].

### Climatic changes between time periods

We estimated annual and seasonal changes of maximum temperature, minimum temperature and precipitation, at three scales: Mexico, the three biogeographic regions (i.e., Nearctic, Neotropical and the Transition Zone) and the 19 biogeographic provinces, using as source data the monthly ANUSPLIN interpolated climate surfaces (grid points).

For estimating changes from *t*_*1*_-1940 to *t*_*2*_-1970 and from *t*_*2*_-1970 to *t*_*3*_-2000 at the country level and the three regions, first, we calculated descriptive statistics using the grid point values of the raster surfaces to summarize temperature and precipitation changes. We also estimated extreme warming and cooling at the 90^th^ and 10^th^ percentile distribution of the grid point values of temperature changes and the median fractional increases and decreases in precipitation. Then, for the 19 provinces, we calculated seasonal changes from *t*_*1*_-1940 to *t*_*2*_-1970 and from *t*_*2*_-1970 to t_*3*_-2000 using the average values of the grid points of the climate surfaces calculated with the software Earth Trends Modeler (ETM) of Idrisi Selva [52]. Seasons were defined as: (i) December, January, February (DJF); (ii) March, April, May (MAM); (iii) June, July, August (JJA); and (iv) September, October, November (SON). Changes in precipitation were estimated in millimeters (mm) and percentage (%), and changes in temperature in Celsius degrees (°C).

To visualize a monotonic upward or downward change through time in the interpolated climate surfaces of annual precipitation (Bio 12), maximum temperature of the warmest month (Bio 05) and minimum temperature of the coldest month (Bio 06), we produced a map showing the consistency of the sign between periods as follows: at each grid point, we obtained the difference between *t*_*2*_-1970 to *t*_*1*_-1940 and between t_*3*_-2000 to *t*_*2*_-1970, if the sign was consistent between these two transitions we classified them either as Positive or Negative, otherwise, we classified them as Not consistent.

Finally, we performed a Pettitt Test for Change-point Detection on annual averages time-series of all individual weather stations used for creating the climate surfaces across the country [58]. The Pettitt test is a non-parametric analysis for detecting inhomogeneities in a time-series and identifying the possible year of breakpoint [59]. Inhomogeneities in climatic time-series may occur owing to climatic factors, or due to instrumental, observer, or positional changes in the stations, and they may distort the signal of climatic change [60]. We used the *pettitt*.*test* function [61] implement in the R software [51].

## Results

### Climate surfaces

Diagnostic measures obtained from ANUSPLIN indicated that model fit of splines varied across historical time slices. The average ratio of the signal to the number of data points was <0.5 for monthly temperatures and precipitation, mostly for *t*_*2*_-1970 and *t*_*3*_*-*2000 (S1 Table in S1 File). For *t*_*1*_-1940, only a small number of weather stations (<900) had available precipitation data. Thus, we eliminated only those stations with higher residual error (>1) to maintain the largest number of stations (>800). The average signal for precipitation was above the permitted threshold (values greater than about half the number of data points), indicating that the number of stations is insufficient to depict the complex spatial patterns of mean precipitation [47]; however, for some months (i.e., from January to May) the ratio signal was acceptable. Therefore, the precipitation surface for period *t*_*1*_-1940 must be used with caution. The monthly average RTMSE for temperatures was below 0.6°C and below 10 mm for precipitation of all three-climate periods.

We found differences between the three time periods in the deseasoned climatic surfaces of monthly precipitation (Wilks' Lambda = 0.295, F_*df* = 24,178478_ = 6249.079, *P <* 0.001), maximum temperature (Wilks' Lambda = 0.419, F_*df* = 24,178478_ = 4050.582, *P <* 0.001) and minimum temperature (Wilks' Lambda = 0.398, F_*df* = 24,178478_ = 4346.174, *P <* 0.001) averaged across all Mexico.

The Pettitt test showed that the number of weather stations holding inhomogeneities varied among the three climatic variables and biogeographic provinces. For maximum temperature, 899 stations out of 4152 (21.7%) presented inhomogeneities, ranging from 10.6–28.2% among provinces (S2 Table in S1 File). These numbers were higher for minimum temperature, for which 36.2% of 5079 presented inhomogeneities. For some provinces inhomogeneities occurred in more than 40% of the stations (S3 Table in S1 File). Inhomogeneities were lower for precipitation (9.4% of 5239 weather stations, ranging from 4–19% among provinces) (S4 Table in S1 File). The breakpoint years–which refers to the estimated year in which an abrupt transition occurred in the time series–were more frequent during the second half of *t*_*2*_*-*1970 and the first half of *t*_*3*_*-*2000 in all three climatic variables and across all provinces (S1, S2 and S3 Figs in S1 File).

### Changes between time periods

The magnitude of change in annual mean temperature (Bio 01) and precipitation (Bio 12) differed between the first and second time periods and between the second and third time periods. At the country level, the mean annual temperature decreased, on average, 0.32°C from *t*_*1*_-1940 to *t*_*2*_-1970, and increased 0.22°C from *t*_*2*_-1970 to *t*_*3*_-2000 (Table 2). This tendency was also consistent across regions, but the Neotropics experienced the greatest change between *t*_*1*_-1940 to *t*_*2*_-1970, (-0.64°C), while the Nearctic did so between *t*_*2*_-1970 and *t*_*3*_-2000 (-0.3°C; Table 2). The largest increases (>1.5°C) from *t*_*2*_-1970 to *t*_*3*_-2000 were found in the desert mountains of northern Mexico and Baja California (S4 Fig in S1 File). Additionally, extreme cooling and warming increased in magnitude since *t*_*2*_-1970 (Table 2).

**Table 2:** Average temperature changes from *t*_*1*_-1940 to *t*_*2*_-1970 and from *t*_*2*_-1970 to *t*_*3*_*-*2000.

Annual mean temperature changes (°C)			
Region	Statistics	*t*_*1*_-1940* to *t*_*2*_-1970	*t*_*2*_-1970 to *t*_*3*_*-*2000
Mexico	Mean	-0.32	0.22
	Median	-0.28	0.30
	10^th^ percentile	-1.10	-0.46
	90^th^ percentile	0.45	0.69
Nearctic	Mean	-0.43	0.30
	Median	-0.33	0.28
	10^th^ percentile	-1.34	-0.26
	90^th^ percentile	0.49	0.89
Transition Zone	Mean	-0.39	0.03
	Median	-0.42	0.06
	10^th^ percentile	-1.33	-0.45
	90^th^ percentile	0.56	0.49
Neotropical	Mean	-0.64	0.06
	Median	-0.70	0.08
	10^th^ percentile	-1.45	-0.43
	90^th^ percentile	0.26	0.48

Summary statistics were computed for Mexico and for three biogeographic regions. To calculate the statistics, we used the grid points (at 30-arc second spatial resolution) that fell within a particular province. Stations used were not corrected for inhomogeneities and densities vary between periods, especially between *t*_*1*_-1940 to *t*_*2*_-1970.

*This period is longer than *t*_*2*_-1970 and *t*_*3*_*-*2000.

**Fig 3:**
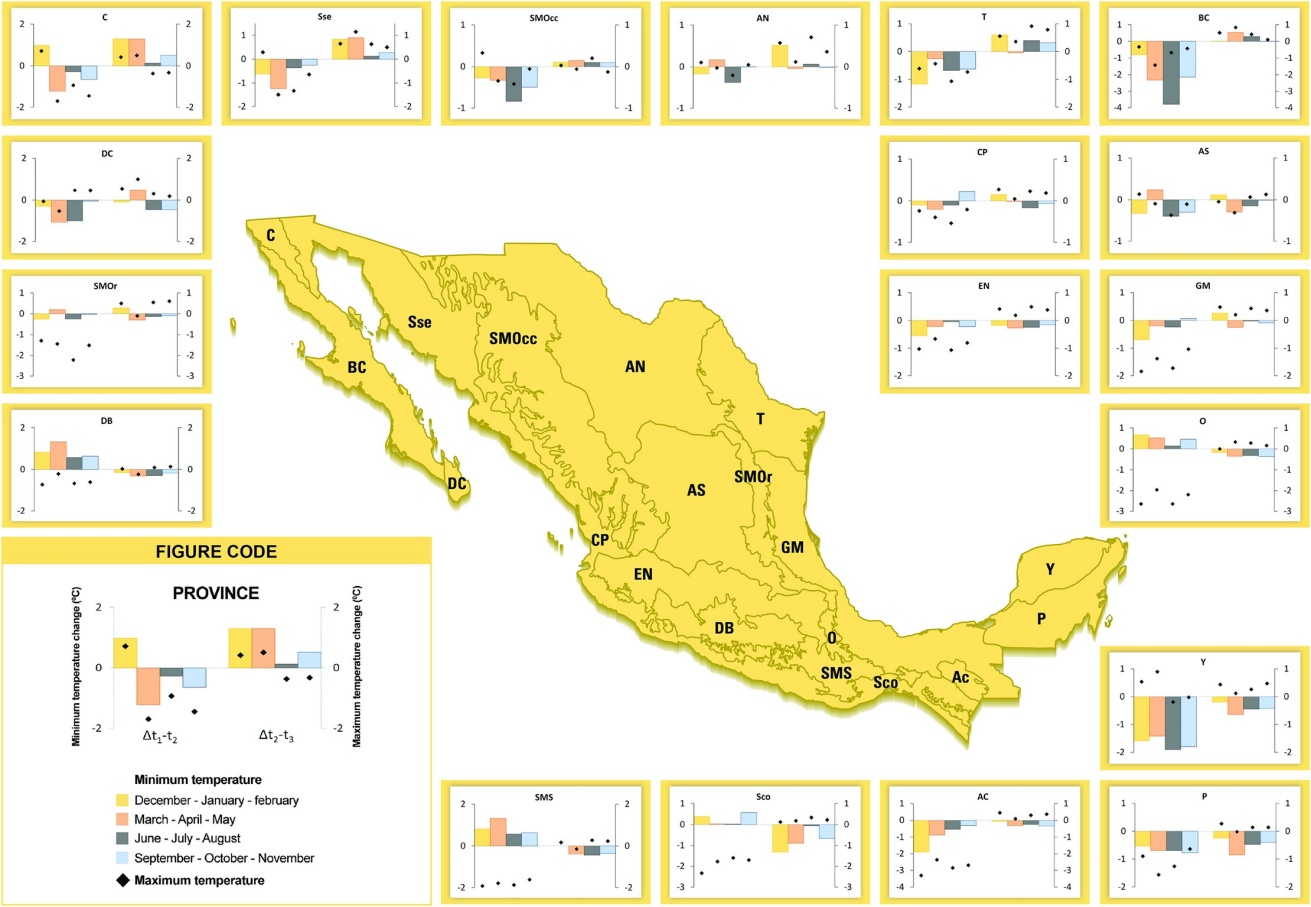
Seasonal change of maximum (♦) and minimum (bars) temperature from t1-1940 to t2-1970 (Δt_*1*_*-t*_*2*_) and from *t*_*2*_-1970 to *t*_*3*_*-*2000 (*Δt*_*2*_*-t*_*3*_) in the biogeographic provinces of Mexico. Negative values indicate a decrease in temperature from the previous period and positive values indicate an increase. DB: Depresión del Balsas, SMOr: Sierra Madre Oriental, DC: Del Cabo, C: California, Sse: Sonorense, SMOcc: Sierra Madre Occidental; AN: Altiplano Norte, T: Tamaulipeca, BC: Baja California, CP: Costa del Pacífico, AS: Altiplano Sur, EN: Eje Neovolcánico, GM: Golfo de México, O: Oaxaca, Y: Yucatán, P: Petén, AC: Altos de Chiapas, Ssc: Soconusco, and SMS: Sierra Madre del Sur. Stations used were not corrected for inhomogeneities and densities vary between periods, especially between *t*_*1*_-1940 to *t*_*2*_-1970. Biogeographic provinces reprinted from [57] under a CC BY license, with permission from [CONABIO], original copyright [1997].

Across Mexico, maximum and minimum temperatures have increased since *t*_*2*_-1970 in most provinces and for almost all seasons, even when at the beginning of the century maximum temperature decreased mainly in the southern provinces of the country (Fig 3). Between *t*_*1*_-1940 and *t*_*2*_-1970, Baja California and Yucatán provinces suffered the greatest changes, with mean annual temperature decreases ranging from almost 2°C to almost 4°C in the summer (Fig 3). Between *t*_*2*_-1970 and *t*_*3*_-2000, there was a tendency of temperature increase in the northern provinces with no consistent change observed in the southern ones (Figs 3 and 5). Within seasons, the pattern was similar to the changes observed in the mean annual temperature (Table 2), as we observed an increase in temperatures throughout periods, particularly for the second half of the 20^th^ century, although the changes in minimum temperature were not consistent between periods. This happened mainly in the biogeographic provinces of Depresión del Balsas, Oaxaca, Sierra Madre del Sur, Oaxaca, and Socunusco. Moreover, the increase observed in the minimum temperature in winter (DJF) and spring (MAM) between *t*_*2*_-1970 to *t*_*3*_-2000 was much larger in the northern provinces than elsewhere in the country (Fig 3).

In general, across Mexico, the total area where positive changes in precipitation occurred during the century is larger than the area with negative changes. However, the magnitude of change in mean precipitation is smaller between *t*_*2*_*-*1970–*t*_*3*_*-*2000 than between *t*_*1*_*-*1940–*t*_*2*_*-*1970 (Table 3). Mean changes in annual precipitation since *t*_*2*_-1970 ranged between 4% increase for the Nearctic to -5% for the Neotropical region. Despite the change in mean precipitation in the Nearctic was positive, median conditions between *t*_*2*_*-*1970–*t*_*3*_*-*2000 have decreased. Changes in precipitation were higher in the Transition Zone and the Neotropical region than in the Nearctic region, with a reduction of precipitation of 3% to 8%, respectively (Table 3).

**Table 3:** Average precipitation changes from *t*_*1*_-1940 to *t*_*2*_-1970 and from *t*_*2*_-1970 to *t*_*3*_*-*2000.

Anual Precipitation (mm)			
Region	Statistics	*t*_*1*_-1940* to *t*_*2*_-1970	*t*_*2*_-1970 to *t*_*3*_*-*2000
Mexico	Mean	123 (16)	-13 (-2)
	Median	48 (8)	1 (0)
	10^th^ percentile	28 (11)	23 (8)
	90^th^ percentile	26 (2)	164 (10)
Nearctic	Mean	3 (1)	18 (4)
	Median	35 (10)	18 (5)
	10^th^ percentile	3 (1)	18 (13)
	90^th^ percentile	41 (7)	-1 (0)
Transition zone	Mean	141 (16)	-12 (-1)
	Median	121 (15)	-13 (-2)
	10^th^ percentile	40 (8)	10 (2)
	90^th^ percentile	262 (21)	-36 (-3)
Neotropical region	Mean	289 (21)	-62 (-5)
	Median	122 (10)	-8 (-1)
	10^th^ percentile	139 (16)	-28 (-4)
	90^th^ percentile	740 (33)	-160 (-8)

Summary statistics are reported as millimeters and percentages (in parenthesis) and were computed for Mexico and for three biogeographic regions. To calculate the statistics, we used the 30-arc gridpoints that fell within a particular province. Stations used were not corrected for inhomogeneities and densities vary between periods, especially between *t*_*1*_-1940 to *t*_*2*_-1970.

*This period is longer than *t*_*2*_-1970 and *t*_*3*_*-*2000.

**Fig 4:**
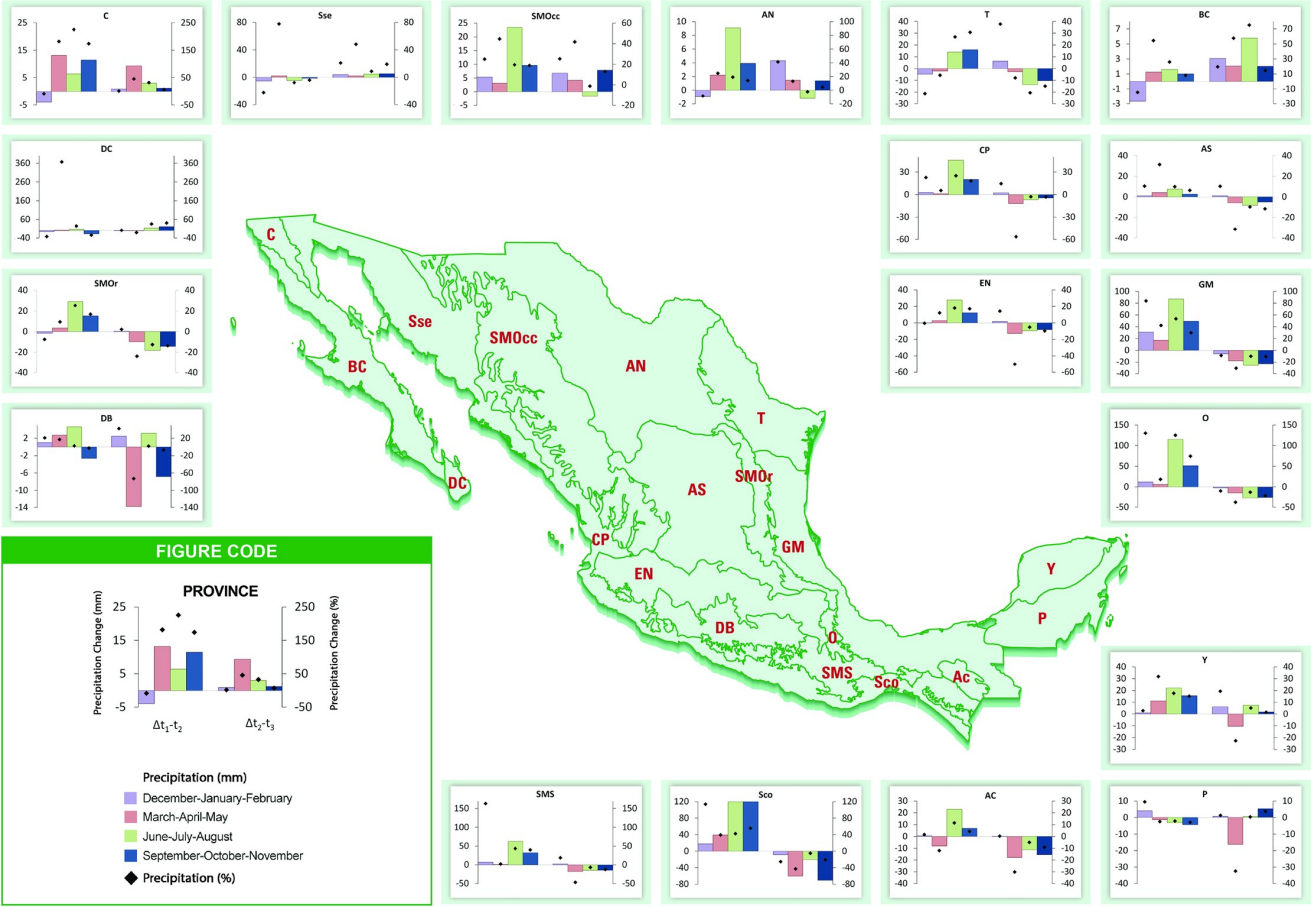
Seasonal precipitation changes in % (♦) and millimeters (bars) from *t*_*1*_-1940 to *t*_*2*_-1970 (*Δt*_*1*_*-t*_*2*_) and from *t*_*2*_-1970 to *t*_*3*_*-*2000 (*Δt*_*2*_*-t*_*3*_) in the biogeographic provinces of Mexico. Negative values indicate a decrease in precipitation from the previous period and positive values indicate an increase. DB: Depresión del Balsas, SMOr: Sierra Madre Oriental, DC: Del Cabo, C: California, Sse: Sonorense, SMOcc: Sierra Madre Occidental; AN: Altiplano Norte, T: Tamaulipeca, BC: Baja California, CP: Costa del Pacífico, AS: Altiplano Sur, EN: Eje Neovolcánico, GM: Golfo de México, O: Oaxaca, Y: Yucatán, P: Petén, AC: Altos de Chiapas, Ssc: Soconusco, and SMS: Sierra Madre del Sur. Stations used were not corrected for inhomogeneities and densities vary between periods, especially between *t*_*1*_-1940 to *t*_*2*_-1970. Biogeographic provinces reprinted from [57] under a CC BY license, with permission from [CONABIO], original copyright [1997].

Precipitation has generally decreased in the tropical provinces of the south and in the mountainous provinces of the Transition Zone, whereas it has increased in the Nearctic provinces for both temporal changes (Fig 4). In several southern biogeographic provinces, the magnitude of precipitation decreased in *t*_*2*_*-*1970–*t*_*3*_*-*2000, mainly during spring (MAM) and autumn (SON; Fig 4). Mean precipitation change was higher from *t*_*1*_-1940 to *t*_*2*_-1970 in five biogeographic provinces (Sierra Madre del Sur, Costa del Pacífico, Oaxaca, Soconusco, and Golfo de México) compared to the mean precipitation change from *t*_*2*_*-*1970 to *t*_*3*_*-*2000. In the remaining provinces, the precipitation change was positive in *t*_*1*_-1940–*t*_*2*_-1970, but magnitude decreased in *t*_*1*_-1940–*t*_*2*_-1970 (Fig 4).

**Fig 5:**
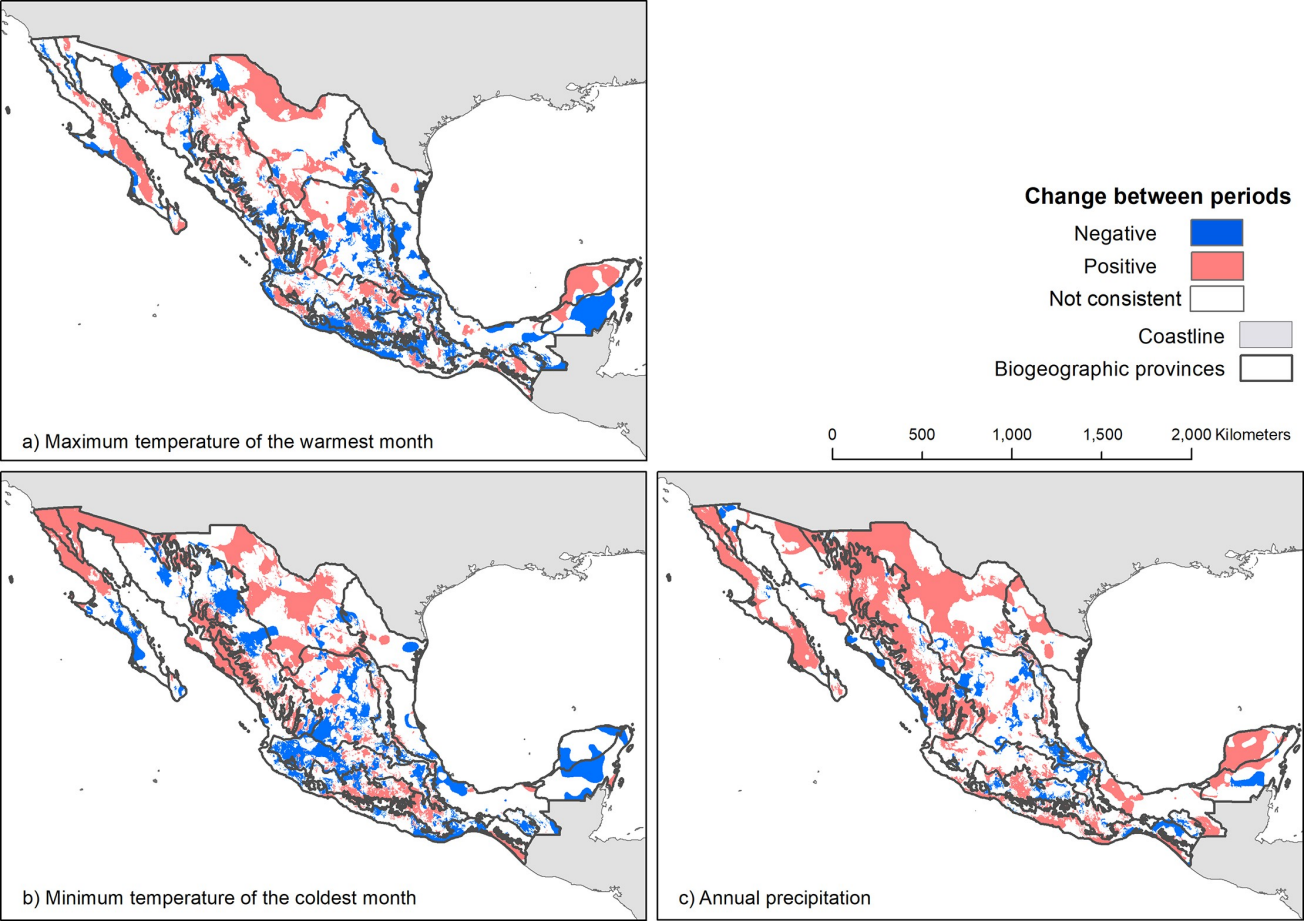
Climate change in Mexico from *t*_*1*_-1910 to *t*_*3*_-2000. Negative and positive areas represent downwards and upwards changes, respectively, in annual precipitation (Bio 12), maximum temperature of the warmest month (Bio 05) and minimum temperature of the coldest month (Bio 06). Blue areas indicate negative signs (decrease) for the difference between *t*_*1*_-1940–*t2*-1970 and *t*_*2*_-1970–*t*_*3*_-2000. Red areas indicate positive signs (increase) for the difference between *t*_*1*_-1940–*t*_*2*_-1970 and *t*_*2*_-1970–*t*_*3*_-2000. White areas indicate that the sign between *t*_*1*_-1940–*t*_*2*_-1970 and t_*2*_-1970–*t*_*3*_-2000 were inconsistent. Biogeographic provinces reprinted from [57] under a CC BY license, with permission from [CONABIO], original copyright [1997].

Monotonic upward or downward change in Bio 12, Bio 05 and Bio 06 occurred over 50% of the interpolated climate surfaces (Fig 5). A consistent reduction in maximum temperature (Bio 05) occurred in 13% of the country, whereas 12% of the country experienced an increase in this parameter. Minimum temperature (Bio 06) decreased monotonically in 26% of the country and consistently increased in 12% of the country. For precipitation (Bio 12), consistent increases and reductions were observed in 18% and 5% of the country, respectively, mainly in the Altiplano Norte, Sierra Madre Occidental, Sonorense, Baja California, and Petén provinces. Positive changes in Bio 06 were particularly consistent in the mountainous regions of the country, at the highest peaks of the Eje Neovolcánico province: Popocatépetl, Iztaccíhuatl and Nevado de Toluca volcanoes. Negative changes for the minimum temperature occurred mainly in Yucatán, Los Altos de Chiapas and Petén provinces (Fig 5). Finally, we identified a consistent increase in Bio 05 in the California, Baja California, Sonorense, Del Cabo, and Altiplano Norte provinces throughout the century (Fig 5).

## Discussion

Climate change is currently the environmental issue of greatest concern worldwide, thus extensive research efforts are underway. Surprisingly, there are very few formal evaluations of historical climate change effects on biodiversity [21]. For this purpose, historical climate interpolations have proven useful to analyze climate variation during the recent past and its effects on biodiversity and agrobiodiversity [21,62]. Here, we developed historic climate interpolated surfaces to evaluate to what extent Mexico and its biogeographic regions and provinces have been exposed to climate change during the 20^th^ and early 21^st^ centuries.

### Significance for ecological studies

Mexico has been projected to maintain medium to high climate stability [36], but, as expected, historically the magnitudes of change have not been geographically uniform across Mexico. Understanding the history of climate change at the regional level can provide useful information for analyzing how climate change impacts biodiversity [62]. Our results show that, on average, minimum and maximum temperatures decreased and precipitation increased countrywide from *t*_*1*_-1940 to *t*_*2*_-1970, whereas from *t*_*2*_-1970 to *t*_*3*_-2000, average minimum and maximum temperatures rose and precipitation decreased. Similar results were obtained for temperature by Pavia *et al*. [19] and Englehart and Douglas [18] from analysis of weather stations’ data. Furthermore, these authors found that warming has not been consistent throughout the country during the 20^th^ century, as we also observed. They reported that cooling occurred mainly in the central and southern provinces of the country in the mid-century (1940–1969). In turn, we observed a decrease of the maximum temperature at the Sierra Madre Oriental and Soconusco provinces from *t*_*1*_-1940 to *t*_*2*_-1970, and an increase since *t*_*2*_-1970 in the Sierra Madre Oriental, Golfo de México, Altos de Chiapas, and Yucatán provinces.

Our analyses demonstrate that the Neotropical region (Costa del Pacífico, Golfo de México, Depresión del Balsas, Oaxaca, Altos de Chiapas, Soconusco, Yucatán, and Petén provinces) has exhibited a more pronounced decline in precipitation, probably as a consequence of an increase in the frequency and intensity of El Niño events in the last two decades [63]. Conversely, precipitation and temperature [19] have shown an increase in some provinces of the north, namely, California, Sonorense, Altliplano Norte, and Tamaulipeca. However, precipitation is counterbalanced by evapotranspiration, and the combined effect of an increase in precipitation and temperature can cause a larger vapor pressure deficit and evaporation, reducing water availability [62], as has been observed in Tamaulipeca, Baja California and Sonorense provinces [64,65]. Arid and semiarid provinces are highly dependent on water availability, regulating net ecosystem productivity [62,66] and agriculture [36,64]. Human population and agriculture have increased in these provinces in the last three decades, exceeding water availability in some areas [63,67].

There are large spatial variations in the projected changes of temperature and precipitation in most of the Altiplano Sur and Eje Neovolcánico provinces, although within the latter we identified an increase in minimum temperature in several high-elevation volcanoes. The rapid retreat of glaciers during the 20^th^ century confirms this observation [68]. The confluence of flora and fauna from the Nearctic and Neotropical regions makes the Transition Zone particularly rich and unique [60]. Many species are endemic to this region and occupy naturally narrow distributional ranges. This condition coupled with the fact that the Transition Zone concentrates the highest human density and largest urban nuclei of the country, its biodiversity is highly vulnerable to the synergistic effects of multiple stressors, including climate change [10]. Furthermore, survival of many species depends on their capacity to keep pace with climate; however, their ability to respond to climatic changes via range shifts are limited due to human-induced obstacles, making them highly vulnerable to the current warming event [36,62].

There are several factors other than global warming that also influence climatic variations through time, for instance, the Pacific Decadal Oscillation, the Atlantic Multidecadal Oscillation and the El Niño Southern Oscillation. At a regional scale, the direct attribution of anthropogenic climate change to the observed patterns is complicated; nonetheless, our results are in line with those obtained by the IPCC, showing increase in temperature since mid-20^th^ century, with greater changes in the north of the country [9]. We also identified a general increase in precipitation throughout the century, although its magnitude decreased between *t*_*2*_*-*1970 and *t*_*3*_*-*2000. This change might be related to the fact that a negative Atlantic Multidecadal Oscillation and a Pacific Decadal Oscillation were observed during the last period [9].

Pervasive climatic change leads to changes in species composition, as has been observed for birds in Mexico [28]. Understanding recent climate and ecological changes are useful to focus research efforts and conservation actions; therefore, the climate changes detected in this study may be useful to establish priorities for conservation. For instance, the Transition Zone includes the Sierra Madre Occidental, Sierra Madre Oriental and Eje Neovolcánico provinces, which harbor diverse types of temperate vegetation, such as oak, pine and cloud forests; all of them, but particularly the latter, are highly vulnerable to climate change [68,69]. The Sierra Madre Oriental harbors the richest coniferous forests in the world [70]. Our results show that this province has been warming since mid-20^th^ century and is projected to be affected by climate change in the future [69,71], thus conservation efforts should be prioritized in this region [70].

Changes in climate are now occurring simultaneously with other environmental disruptions. It will not be possible to fully understand biodiversity responses to climate change without considering the interactions with other components of environmental change [72]. Species responses will depend on their exposure and sensitivity to other human-induced pressures, their inherent capacity to adapt to new conditions, the magnitude and speed of environmental changes, and time lags in their responses [1,10,62]. This work aims to serve as a baseline for improving our knowledge regarding historic climate change in Mexico in a spatially-explicit fashion, to identify specific areas where climate change is occurring and to identify its direction and magnitude.

In sum, climate change is occurring unevenly in Mexico. Provinces in the Nearctic region showed a higher and more consistent warming over time than provinces from the Transition Zone and Neotropical region. Precipitation has also generally increased more consistently in northern provinces over the whole period, whereas it tended to decrease in most of the Neotropical region and Transition Zone between *t*_*2*_*-*1970 and *t*_*3*_-2000. Nonetheless, it is important to bear in mind that the first period (*t1*-1940) holds higher uncertainty than the other two given the lower number of weather stations used to produce the climatic surfaces. Finally, this information can be used to improve regional projections of future climate impacts on biodiversity, which would provide scientists and authorities with more reliable data and information for making better decisions in the face of climate change.

### Limitation of the analysis

We have generated three sets of climatic surfaces for Mexico with error estimates based on withheld data across the country [42] comparable to errors obtained in two other climate surface datasets developed for the country for different time frames [15,17]. However, it is important to mention the main shortcomings in this type of data and analysis for a better use of them. First, it has been demonstrated that long-term station records that are used to interpolate climate surfaces generally suffer from incompleteness and inconsistencies through time caused by equipment failure, replacement or station relocation [40,73], limiting their use for calculating multi-decadal climate trends [72,74]. This is the case for the data that we used for generating these climatic surfaces. Our results of the Pettitt test indicate that a considerable proportion of the stations presented inhomogeneities along the time-series, especially for minimum temperature (see S1 File). Furthermore, the breakpoint years occurred mostly during the second half of *t*_*2*_*-*1970 and the first half of *t*_*3*_*-*2000 (S1, S2, S3 Figs in S1 File). To know how much of these inhomogeneities are due to climatic or non-climatic factors requires further investigation. Regardless, it is likely that non-climatic variations may have influenced to some extent the observed changes between these periods. Consequently, these non-climatic variations also increase uncertainty in ecological studies [75]; hence, additional sources, such as remote sensing-based climate products can be used to reduce uncertainty, e.g., [76].

Second, climate surfaces for *t*_*1*_-1940 were interpolated for a longer period but with fewer weather stations, thus are more uncertain than the climate surfaces for the other two periods [17,40]. We advise caution when using the surfaces from this period. Finally, there is a geographical bias in the location of weather stations in the three periods, since density of stations was higher in or nearby human settlements and lower in remote locations, some of which can be important for biodiversity analyses [74,77]. This geographical bias has implications in the spatial structure of error and uncertainty, particularly in areas with low station density [75], where scarcity of stations impedes to capture the effect of terrain complexity in climate variability [78]. Considering all these limitations in the data used for the construction of the climatic surfaces, the use of them and the interpretation of the results should be made with caution.

## Supporting information

S1 File ANUSPLIN statistics and additional spatial distribution of climate data. This file contains the http of a kmz with the distribution of weather stations across Mexico and ANUSPLIN statistics. We also included results of the Pettitt test for change-point detection in precipitation, maximum and minimum temperature stations, and three figures that describe the frequency distribution of the detected breakpoints years. Finally, we share a map showing extremes of warming.
(DOCX)
