# Supplementary material for: One hundred years of climate change in Mexico
Source: PLoS One. 2020 Jul 16;15(7):e0209808. doi: 10.1371/journal.pone.0209808 (PMC7365465; doi:10.1371/journal.pone.0209808)
Supplement: S1 File — This file contains the http of a kmz with the distribution of weather stations across Mexico and ANUSPLIN statistics. We also included results of the Pettitt test for change-point detection in precipitation, maximum and minimum temperature stations, and three figures that describe the frequency distribution of the detected breakpoints years. Finally, we share a map showing extremes of warming. (DOCX) [file pone.0209808.s001.docx]

**Supporting information**.

**S1.** KMZ that includes several aspects of Mexico weather station, such as period of record for each station <https://smn.cna.gob.mx/tools/RESOURCES/estacion/EstacionesClimatologicas.kmz>

**S1 Table.** The average signal ratio to the total number of observations and the root mean square error (RTMSE) for the spline model of monthly temperatures and precipitation surfaces.

|  | I | | | | | | II | | | | | | III | | | | | |
| --- | --- | --- | --- | --- | --- | --- | --- | --- | --- | --- | --- | --- | --- | --- | --- | --- | --- | --- |
|  | Minimum temperature (°C) | | Maximum temperature (°C) | | Precipitation (mm) | | Minimum temperature (°C) | | Maximum temperature (°C) | | Precipitation (mm) | | Minimum temperature (°C) | | Maximum temperature (°C) | | Precipitation (mm) | |
| Month | Ratio | RTMSE | Ratio | RTMSE | Ratio | RTMSE | Ratio | RTMSE | Ratio | RTMSE | Ratio | RTMSE | Ratio | RTMSE | Ratio | RTMSE | Ratio | RTMSE |
| 1 | 0.35 | 0.67 | 0.43 | 0.57 | 0.62 | 4.65 | 0.21 | 0.63 | 0.18 | 0.60 | 0.28 | 4.08 | 0.22 | 0.69 | 0.20 | 0.57 | 0.26 | 4.00 |
| 2 | 0.27 | 0.59 | 0.43 | 0.54 | 0.62 | 4.38 | 0.22 | 0.58 | 0.19 | 0.57 | 0.28 | 3.15 | 0.21 | 0.62 | 0.21 | 0.53 | 0.26 | 2.74 |
| 3 | 0.20 | 0.55 | 0.55 | 0.58 | 0.61 | 3.98 | 0.21 | 0.62 | 0.21 | 0.64 | 0.28 | 3.21 | 0.20 | 0.66 | 0.22 | 0.60 | 0.28 | 2.63 |
| 4 | 0.22 | 0.56 | 0.56 | 0.57 | 0.62 | 4.58 | 0.23 | 0.61 | 0.21 | 0.63 | 0.29 | 3.92 | 0.21 | 0.64 | 0.22 | 0.62 | 0.27 | 3.15 |
| 5 | 0.26 | 0.55 | 0.50 | 0.62 | 0.57 | 7.05 | 0.23 | 0.59 | 0.23 | 0.66 | 0.33 | 6.41 | 0.20 | 0.63 | 0.23 | 0.64 | 0.28 | 5.45 |
| 6 | 0.25 | 0.49 | 0.44 | 0.59 | 0.85 | 8.55 | 0.23 | 0.52 | 0.24 | 0.63 | 0.36 | 12.20 | 0.20 | 0.57 | 0.22 | 0.58 | 0.31 | 10.80 |
| 7 | 0.30 | 0.49 | 0.44 | 0.55 | 0.74 | 10.8 | 0.21 | 0.48 | 0.25 | 0.62 | 0.35 | 15.30 | 0.19 | 0.54 | 0.21 | 0.57 | 0.33 | 12.80 |
| 8 | 0.33 | 0.49 | 0.46 | 0.54 | 0.75 | 11.5 | 0.21 | 0.47 | 0.23 | 0.61 | 0.36 | 15.10 | 0.17 | 0.51 | 0.21 | 0.56 | 0.34 | 12.60 |
| 9 | 0.29 | 0.46 | 0.46 | 0.49 | 0.80 | 12.7 | 0.22 | 0.45 | 0.22 | 0.56 | 0.37 | 14.80 | 0.18 | 0.50 | 0.20 | 0.52 | 0.31 | 13.00 |
| 10 | 0.25 | 0.51 | 0.48 | 0.49 | 0.73 | 7.74 | 0.23 | 0.53 | 0.21 | 0.59 | 0.36 | 9.07 | 0.21 | 0.57 | 0.20 | 0.54 | 0.31 | 8.12 |
| 11 | 0.24 | 0.62 | 0.41 | 0.52 | 0.73 | 4.94 | 0.24 | 0.58 | 0.20 | 0.56 | 0.35 | 5.01 | 0.21 | 0.63 | 0.18 | 0.52 | 0.29 | 3.89 |
| 12 | 0.29 | 0.64 | 0.41 | 0.54 | 0.72 | 4.28 | 0.23 | 0.62 | 0.20 | 0.59 | 0.33 | 4.23 | 0.21 | 0.68 | 0.19 | 0.55 | 0.29 | 3.76 |
| Average | 0.27 | 0.55 | 0.47 | 0.55 | 0.71 | 7.71 | 0.22 | 0.56 | 0.21 | 0.60 | 0.34 | 9.35 | 0.20 | 0.61 | 0.21 | 0.57 | 0.30 | 8.04 |

**S2 Table.** Results of the Pettitt test for Change-point Detection for annual maximum temperature. The table shows the number of weather stations in each biogeographic province, and the number and percentage of weather stations with inhomogeneities and no inhomogeneities detected at p < 0.05.

|  | Maximun temperature | | | | |
| --- | --- | --- | --- | --- | --- |
| Province | No. stations | With inhomogeneities | % | With no inhomogeneities | % |
| Altiplano Norte | 240 | 29 | 12.1 | 211 | 87.9 |
| Altiplano Sur | 541 | 138 | 25.5 | 403 | 74.5 |
| Baja California | 117 | 33 | 28.2 | 84 | 71.8 |
| California | 46 | 9 | 19.6 | 37 | 80.4 |
| Costa del Pacifico | 460 | 111 | 24.1 | 349 | 75.9 |
| Del Cabo | 43 | 11 | 25.6 | 32 | 74.4 |
| Depresion del Balsas | 250 | 47 | 18.8 | 203 | 81.2 |
| Eje Volcanico | 740 | 171 | 23.1 | 569 | 76.9 |
| Golfo de Mexico | 499 | 113 | 22.6 | 386 | 77.4 |
| Los Altos de Chiapas | 49 | 12 | 24.5 | 37 | 75.5 |
| Oaxaca | 36 | 10 | 27.8 | 26 | 72.2 |
| Peten | 64 | 8 | 12.5 | 56 | 87.5 |
| Sierra Madre del Sur | 155 | 23 | 14.8 | 132 | 85.2 |
| Sierra Madre Occidental | 252 | 37 | 14.7 | 215 | 85.3 |
| Sierra Madre Oriental | 183 | 48 | 26.2 | 135 | 73.8 |
| Soconusco | 15 | 3 | 20.0 | 12 | 80.0 |
| Sonorense | 216 | 52 | 24.1 | 164 | 75.9 |
| Tamaulipeca | 142 | 15 | 10.6 | 127 | 89.4 |
| Yucatan | 104 | 29 | 27.9 | 75 | 72.1 |
| Total | 4152 | 899 | 21.7 | 3253 | 78.3 |

**S3 Table.** Results of the Pettitt test for Change-point Detection for annual minimum temperature. The table shows the number of weather stations in each biogeographic province, and the number and percentage of weather stations with inhomogeneities and no inhomogeneities detected at p < 0.05.

|  | Minimum temperature | | | | |
| --- | --- | --- | --- | --- | --- |
| Province | No. stations | With inhomogeneities | % | With no inhomogeneities | % |
| Altiplano Norte | 313 | 90 | 28.8 | 223 | 71.2 |
| Altiplano Sur | 617 | 266 | 43.1 | 351 | 56.9 |
| Baja California | 140 | 49 | 35.0 | 91 | 65.0 |
| California | 56 | 14 | 25.0 | 42 | 75.0 |
| Costa del Pacifico | 577 | 196 | 34.0 | 381 | 66.0 |
| Del Cabo | 46 | 20 | 43.5 | 26 | 56.5 |
| Depresion del Balsas | 294 | 100 | 34.0 | 194 | 66.0 |
| Eje Volcanico | 870 | 340 | 39.1 | 530 | 60.9 |
| Golfo de Mexico | 641 | 245 | 38.2 | 396 | 61.8 |
| Los Altos de Chiapas | 57 | 27 | 47.4 | 30 | 52.6 |
| Oaxaca | 52 | 21 | 40.4 | 31 | 59.6 |
| Peten | 76 | 26 | 34.2 | 50 | 65.8 |
| Sierra Madre del Sur | 202 | 61 | 30.2 | 141 | 69.8 |
| Sierra Madre Occidental | 307 | 83 | 27.0 | 224 | 73.0 |
| Sierra Madre Oriental | 217 | 105 | 48.4 | 112 | 51.6 |
| Soconusco | 18 | 5 | 27.8 | 13 | 72.2 |
| Sonorense | 298 | 85 | 28.5 | 213 | 71.5 |
| Tamaulipeca | 169 | 50 | 29.6 | 119 | 70.4 |
| Yucatan | 129 | 57 | 44.2 | 72 | 55.8 |
| Total | 5079 | 1840 | 36. 2 | 3239 | 63.8 |

**S4 Table.** Results of the Pettitt test for Change-point Detection for precipitation. The table shows the number of weather stations in each biogeographic province, and the number and percentage of weather stations with inhomogeneities and no inhomogeneities detected at p < 0.05.

|  | Precipitation | | | | |
| --- | --- | --- | --- | --- | --- |
| Province | No. stations | With inhomogeneities | % | With no inhomogeneities | % |
| Altiplano Norte | 324 | 29 | 9.0 | 295 | 91.0 |
| Altiplano Sur | 622 | 53 | 8.5 | 569 | 91.5 |
| Baja California | 144 | 17 | 11.8 | 127 | 88.2 |
| California | 56 | 4 | 7.1 | 52 | 92.9 |
| Costa del Pacifico | 598 | 54 | 9.0 | 544 | 91.0 |
| Del Cabo | 50 | 2 | 4.0 | 48 | 96.0 |
| Depresion del Balsas | 305 | 31 | 10.2 | 274 | 89.8 |
| Eje Volcanico | 905 | 103 | 11.4 | 802 | 88.6 |
| Golfo de Mexico | 666 | 63 | 9.5 | 603 | 90.5 |
| Los Altos de Chiapas | 58 | 11 | 19.0 | 47 | 81.0 |
| Oaxaca | 53 | 3 | 5.7 | 50 | 94.3 |
| Peten | 80 | 10 | 12.5 | 70 | 87.5 |
| Sierra Madre del Sur | 205 | 17 | 8.3 | 188 | 91.7 |
| Sierra Madre Occidental | 315 | 18 | 5.7 | 297 | 94.3 |
| Sierra Madre Oriental | 232 | 28 | 12.1 | 204 | 87.9 |
| Soconusco | 20 | 2 | 10.0 | 18 | 90.0 |
| Sonorense | 303 | 21 | 6.9 | 282 | 93.1 |
| Tamaulipeca | 174 | 12 | 6.9 | 162 | 93.1 |
| Yucatan | 129 | 12 | 9.3 | 117 | 90.7 |
| Total | 5239 | 490 | 9.4 | 4749 | 90.6 |

**S1 Fig.** **Frequency distribution of the years where breakpoints were detected for weather stations with inhomogeneities in the biogeographic provinces for maximum temperature**. Bold brown lines mark the three periods analyzed in this study. a. Provinces of the Nearctic region. b. Provinces of the Transition zone, c. Provinces of the Neotropical region.


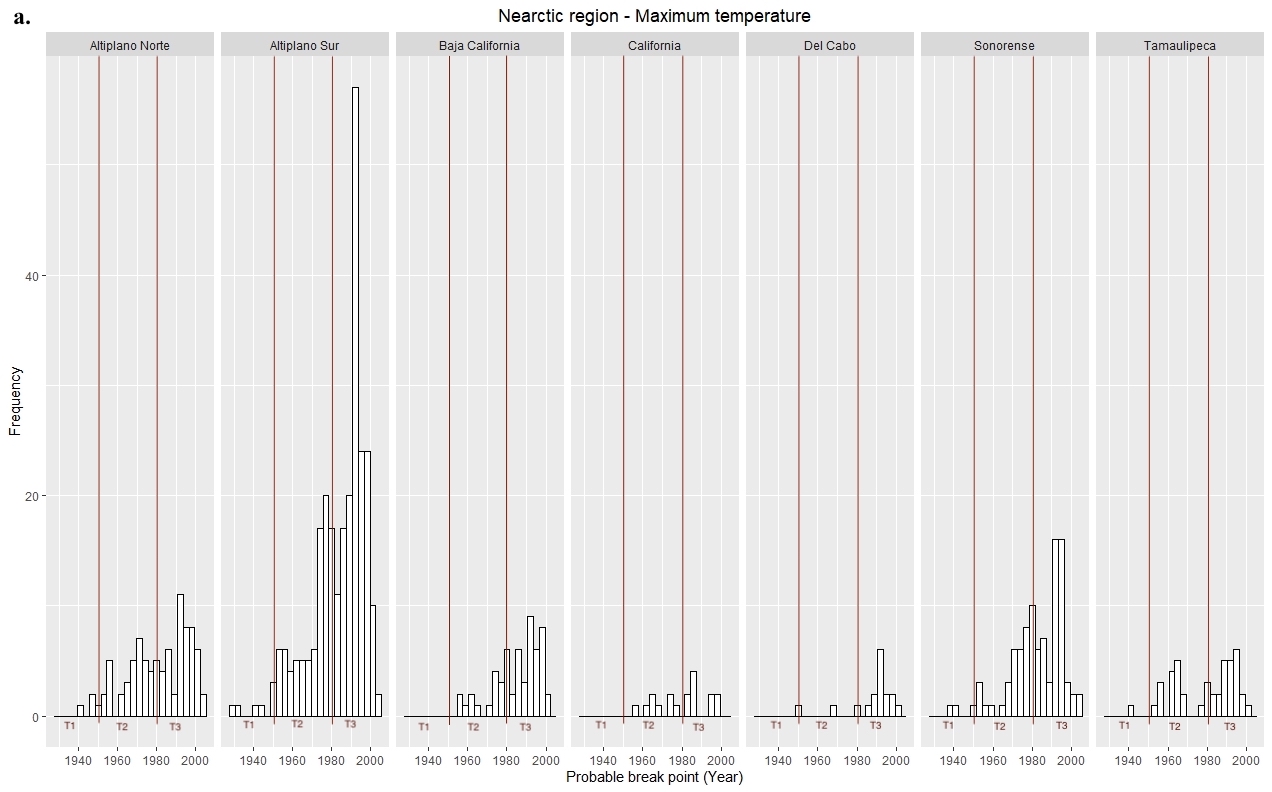


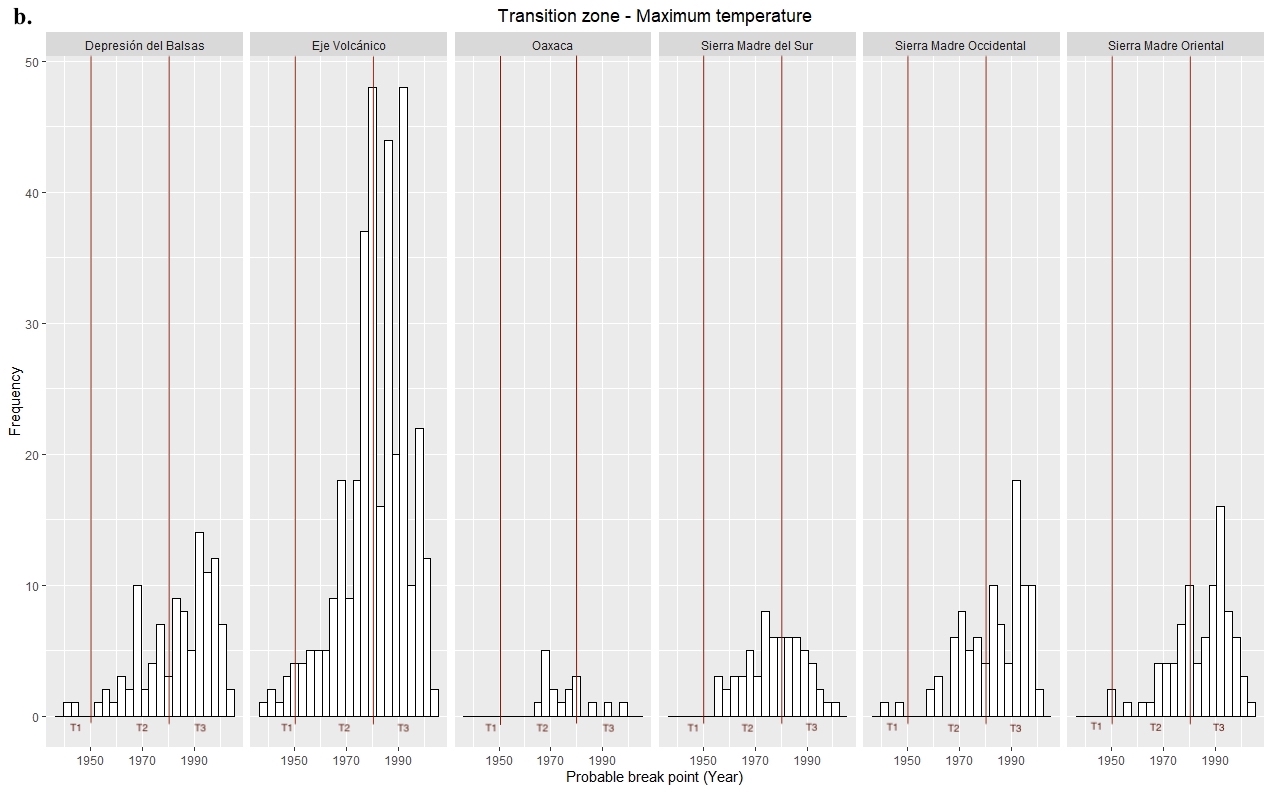


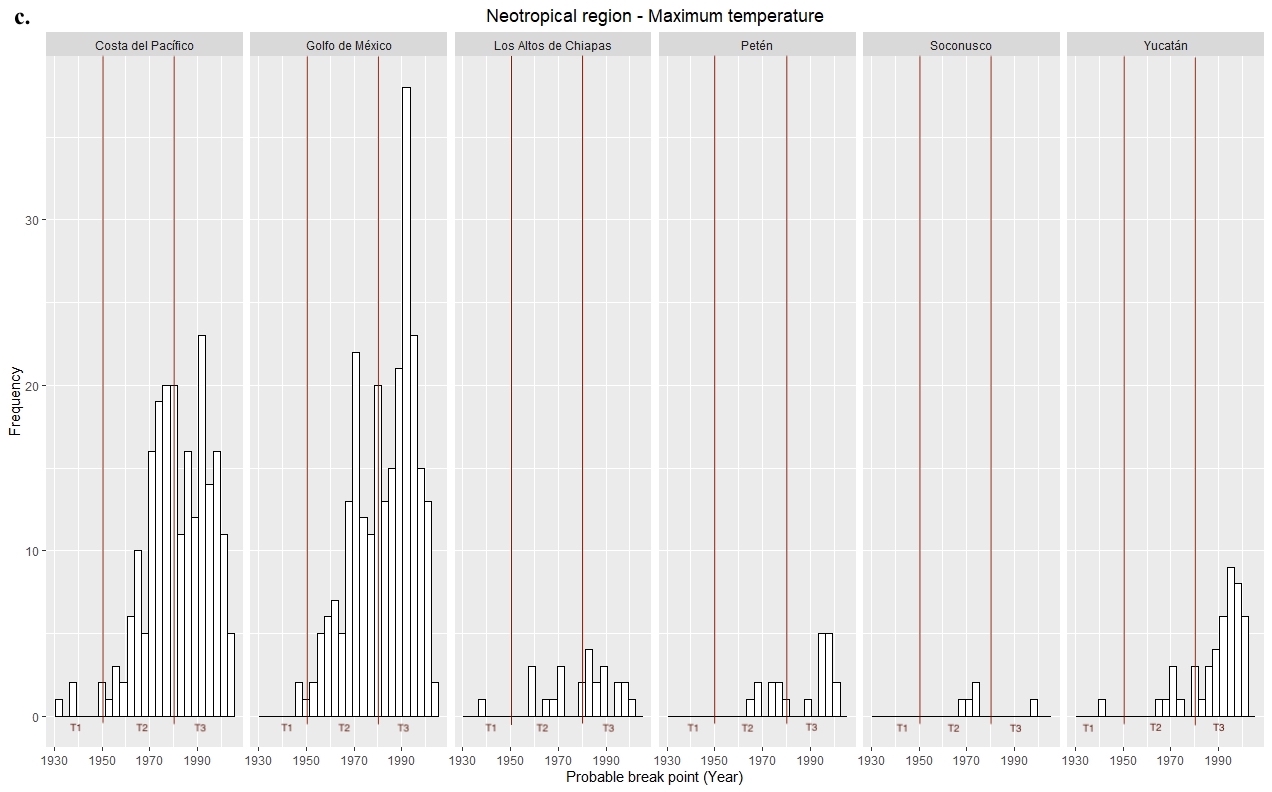


**S2 Fig.** **Frequency distribution of the years where breakpoints were detected for weather stations with inhomogeneities in the biogeographic provinces for minimum temperature**. Bold brown lines mark the three periods analyzed in this study. a. Provinces of the Nearctic region. b. Provinces of the Transition zone, c. Provinces of the Neotropical region.


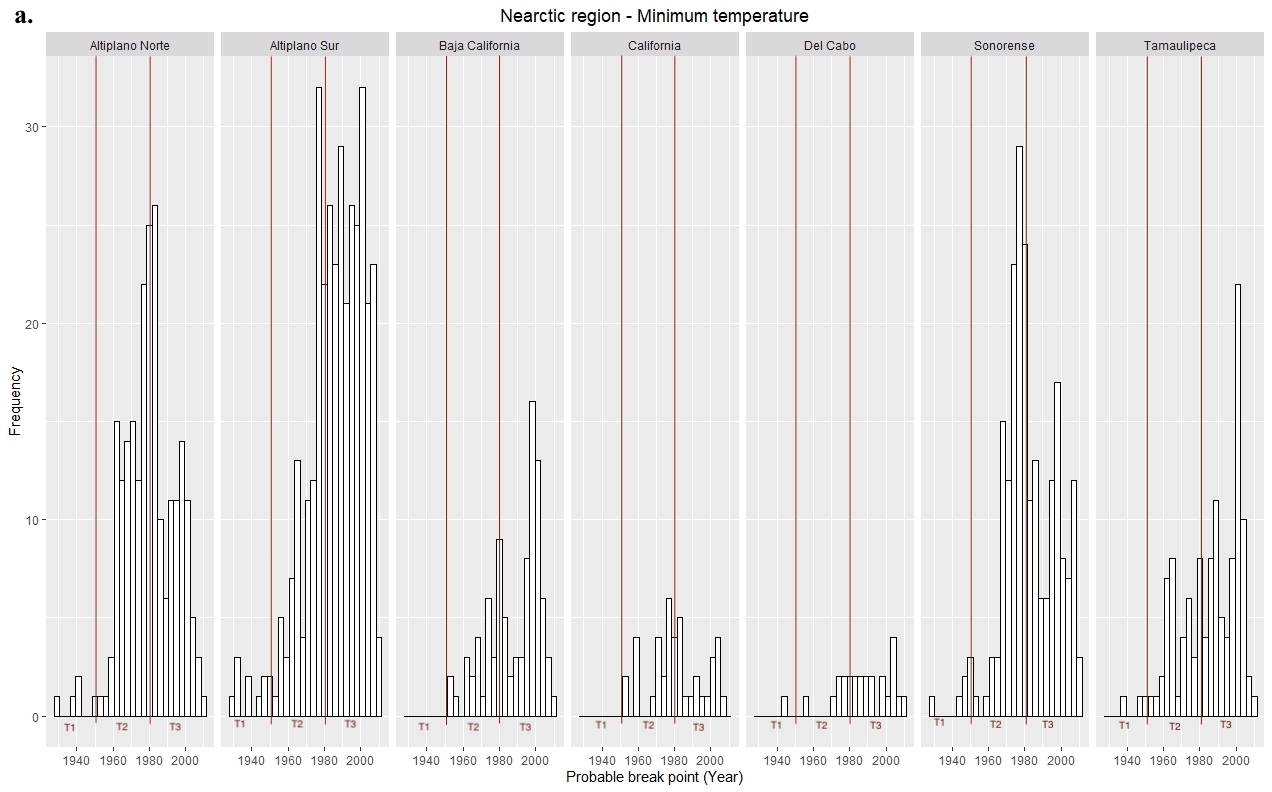

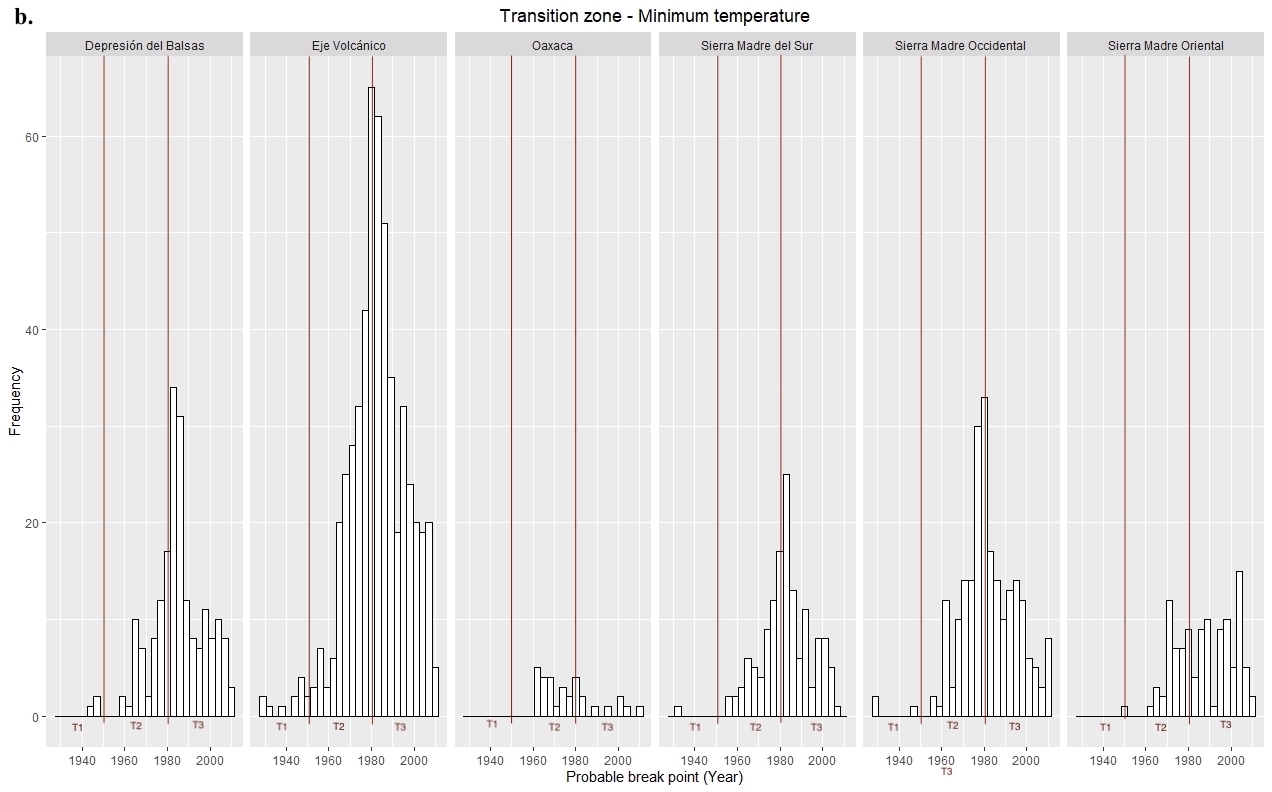

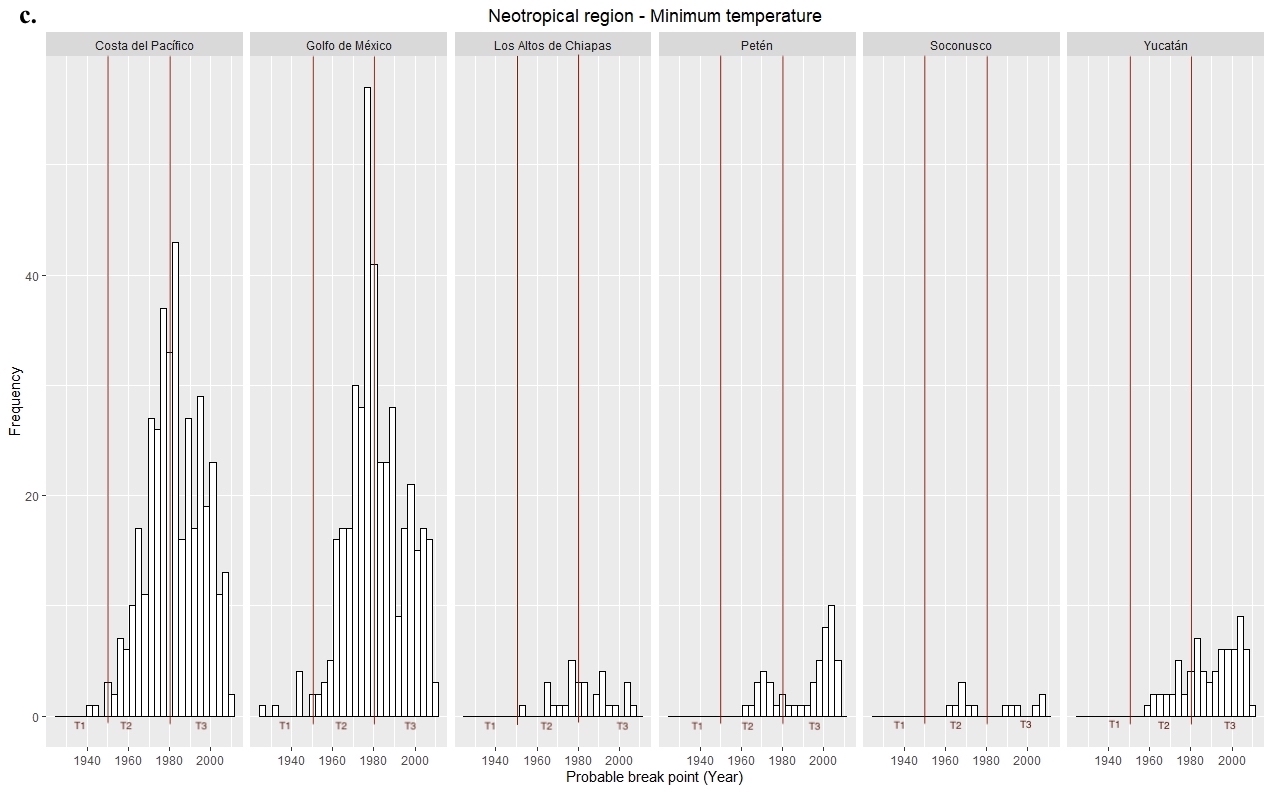


**S3 Fig.** **Frequency distribution of the years where breakpoints were detected for weather stations with inhomogeneities in the biogeographic provinces for precipitation**. Bold brown lines mark the three periods analyzed in this study. a. Provinces of the Nearctic region. b. Provinces of the Transition zone, c. Provinces of the Neotropical region.


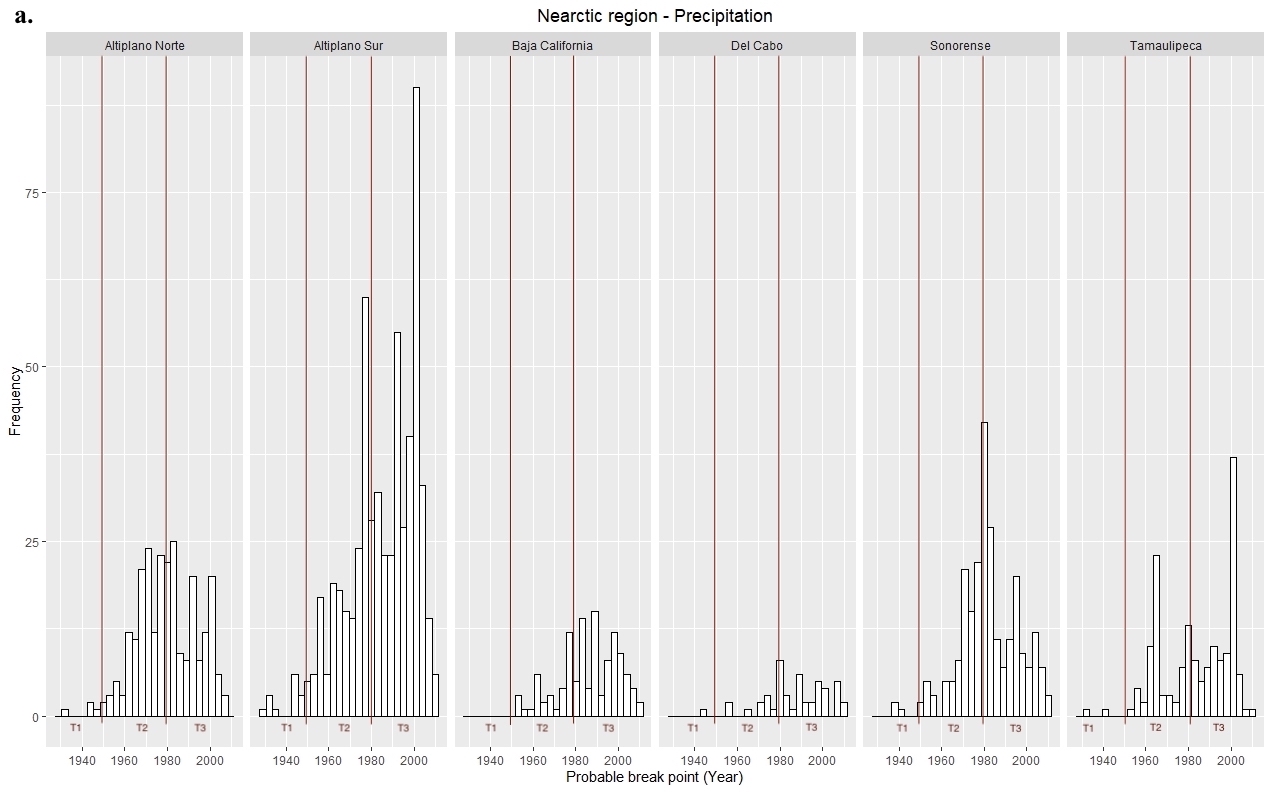


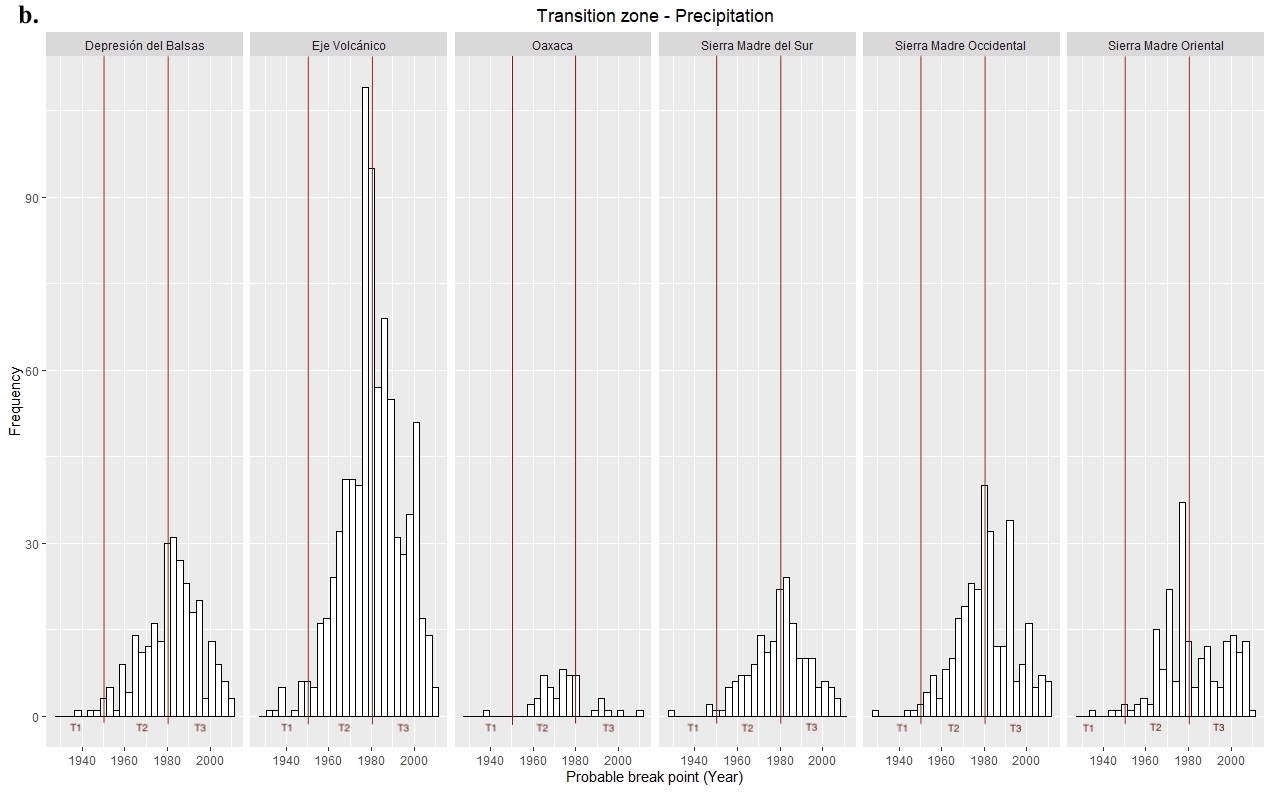


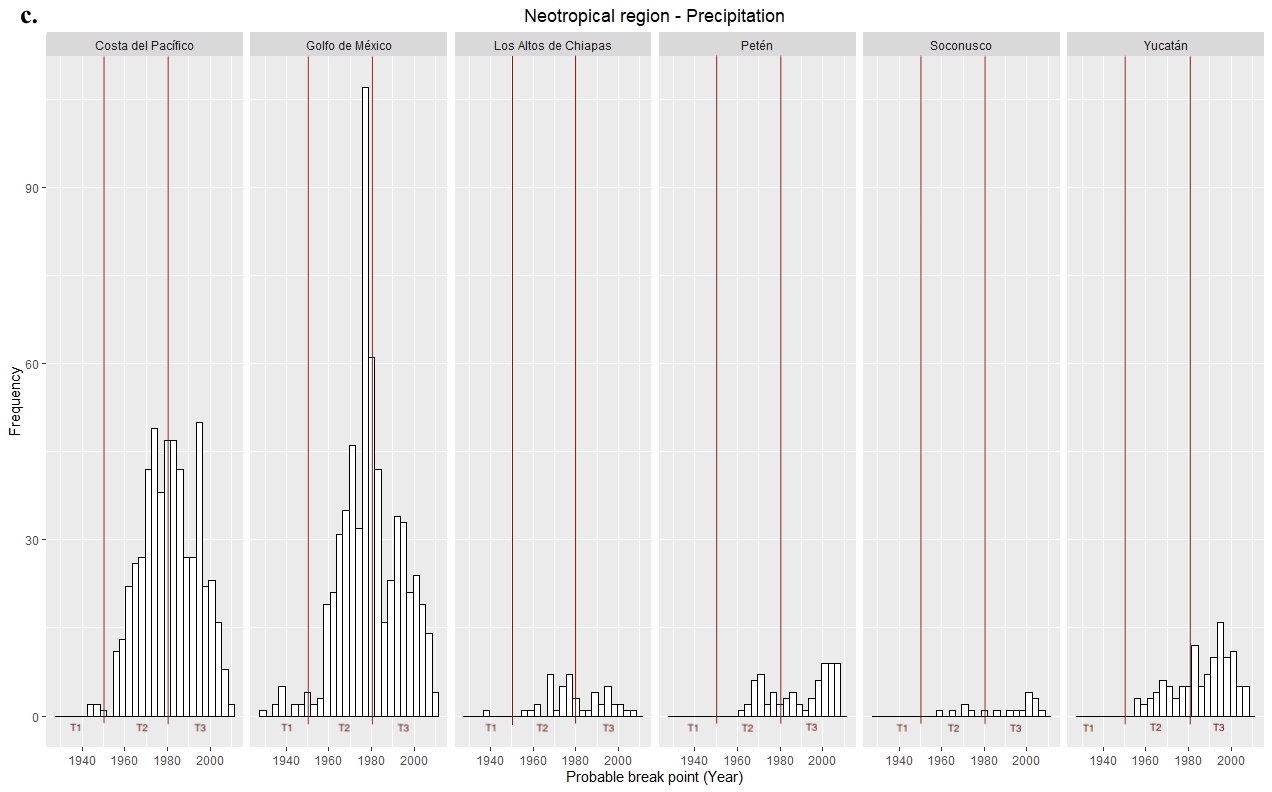


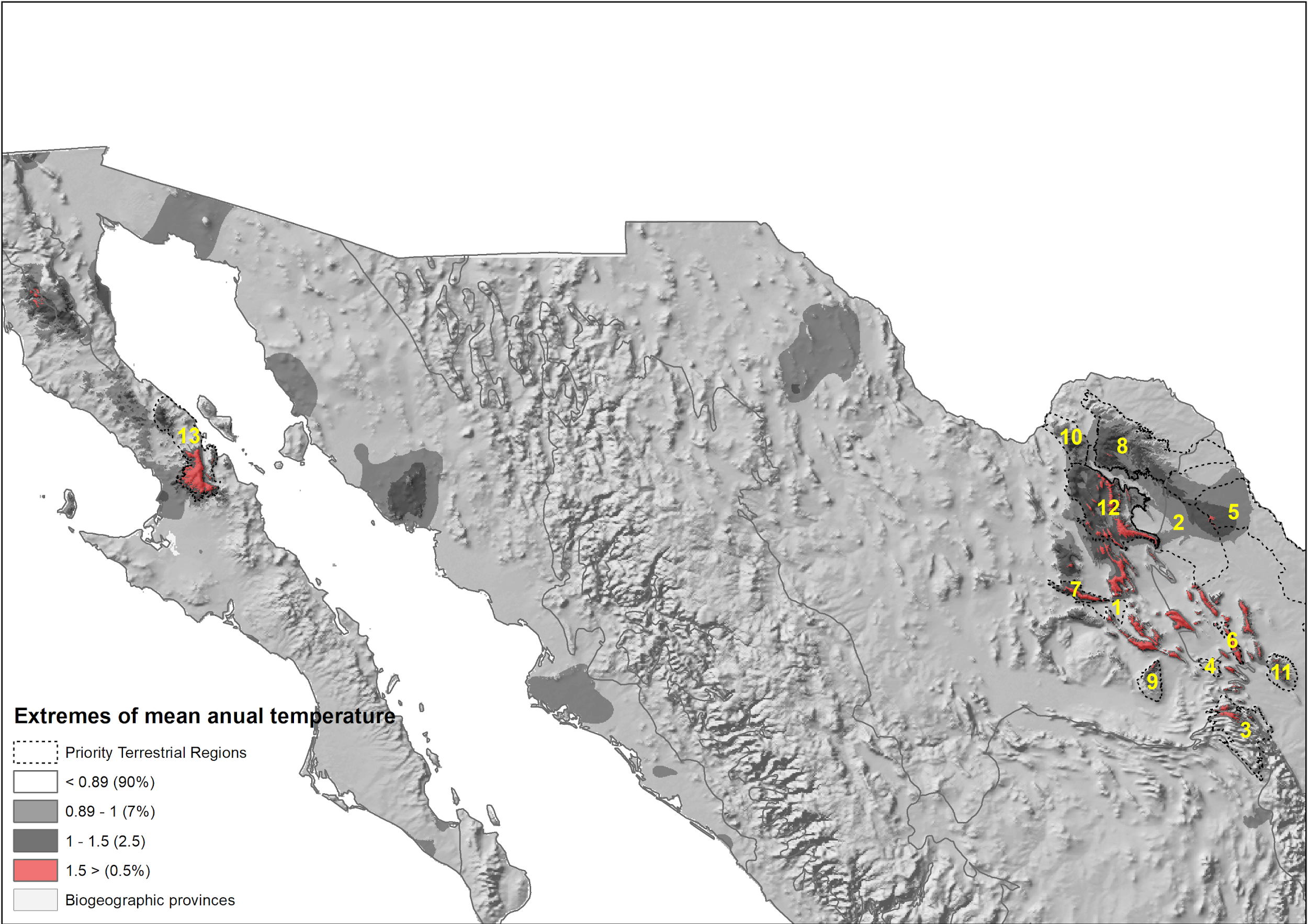


**S4 Fig.** **Extremes of warming are concentrated in the northern east of the Altiplano Norte and Tamaulipeca biogeographic provinces.** Fractional area is representent in percentage. 1. Cuatro Ciénegas; 2. Cuenca del río Sabinas; 3. El Potosí-Cumbres de Monterrey; 4. La Popa; 5. Matorral tamaulipeco del bajo río Bravo; 6. Sierra Bustamante; 7. Sierra de La Madera; 8. Sierra El Burro-río San Rodrigo; 9. Sierra La Paila; 10. Sierra Maderas del Carmen; 11. Sierra Picachos; 12. Sierras La Encantada-Santa Rosa and 13. Sierras La Libertad-La Asamblea.
